# Pharmacy Student Perceptions of a Virtual Pharmacogenomics Activity

**DOI:** 10.3390/healthcare10020286

**Published:** 2022-02-01

**Authors:** Darrow Thomas, John A. Soldner, Cheryl D. Cropp, Jennifer Beall

**Affiliations:** 1 Central Alabama Veterans Health Care System, 215 Perry Hill Rd, Montgomery, AL 36109, USA; Dgerrell@ymail.com; 2 Department of Genetics, The University of Alabama at Birmingham, 720 20th Street South, Birmingham, AL 35294, USA; jsoldner@uab.edu; 3 Department of Pharmaceutical, Social, and Administrative Sciences; Samford University McWhorter School of Pharmacy, 800 Lakeshore Drive, Birmingham, AL 35229, USA; ccropp@samford.edu; 4 Department of Pharmacy Practice; Samford University McWhorter School of Pharmacy, 800 Lakeshore Drive, Birmingham, AL 35229, USA

**Keywords:** pharmacogenomics, learning activity, pharmacy education, asynchronous learning, virtual learning, student survey

## Abstract

Pharmacogenomics (PGx) utilizes a patient’s genome to guide drug treatment and dosing. The Accreditation Council for Pharmacy Education (ACPE) included PGx as a critical content area. Pharmacists are increasingly involved in providing this service, which necessitates training. Second-year pharmacy students at Samford University McWhorter School of Pharmacy have didactic training in the principles of PGx and managing drug therapy using PGx data. A clinical skills lab activity was developed to reinforce these principles and allow students to navigate resources to develop and communicate recommendations for drug therapy. The activity was initially planned as synchronous, but transitioned to asynchronous when students began remote learning in the spring of 2020 due to the COVID-19 pandemic. The investigators sought students’ perceptions of the PGx lab activity and the delivery of its content via a virtual format. This study gathered data from an anonymous, voluntary student survey through Samford University’s course management system, Canvas, in the spring of 2020 soon after completion of the virtual PGx learning activity. The investigators’ goal is to obtain the information and insights obtained from the students who participated in the PGx lab activity to provide guidance for the improvement of their PGx lab activity and for other schools of pharmacy to deliver a PGx lab activities using nontraditional teaching methodologies.

## 1. Introduction

Pharmacogenomics (PGx) studies the relationship between a patient’s genetic variations and how those variations impact the response to medication [1]. This field has developed rapidly since the completion of the Human Genome Project in 2003 [2]. President Barack Obama launched the Precision Medicine Initiative in 2016 to advance medicine from a population-focused approach to a patient-focused one [3].

Patients can now receive a report on their pharmacogenetic variants through direct-to-consumer products, such as 23andMe^®^ [4]. Resources, such as PGx information in drug labeling, are available for those pharmacists who use PGx to manage medication therapy [5]. Online resources are also available including the Clinical Pharmacogenetics Implementation Consortium (CPIC^®^) and The Pharmacogenomics Knowledgebase (PharmGKB) [1,6,7,8].

While there is support for the field and resources available, its implementation into curricula and practice has not been as swift. The 2007–2008 Argus Commission released updated policy statements on biotechnology, which included personalized medicine [9]. The statements were that pharmacy curricula must address advances in these fields, to include genetics/genomics, and that faculty development is needed to prepare them to lead and contribute to this field. In 2015, the American Society of Health-System Pharmacists published a position statement on the role of pharmacists in PGx [10]. This statement originated from the belief that PGx testing can improve outcomes related to medications and delineate pharmacists’ responsibilities and functions in this field. Additionally, the Accreditation Council for Pharmacy Education (ACPE) included PGx as one of the content areas “viewed as central to a contemporary, high-quality pharmacy education” [11].

PGx and its applications are viewed as important and beneficial to patients, yet confidence in its application remains lacking. In a survey of health sciences and other university students, Siamoglou and colleagues found that the students held positive attitudes towards PGx and its benefits on disease management, drug efficacy, and reduction of adverse effects [12]. Zawiah and colleagues found strong support from pharmacy and medical students of PGx testing to help to decrease adverse events, optimize drug dosing and improve drug efficacy [13]. The majority of these students did not agree that they were competent to discuss PGx information with other providers, or that they could accurately apply PGx test results. The authors concluded that there is a need to improve knowledge and better prepare pharmacy and medical students to apply PGx in practice.

Samford University McWhorter School of Pharmacy is a private school in the Southeastern United States. A PGx activity was developed as part of a required skills lab course. This lab course is the third in a six-course sequence that allows for the teaching, practice, and assessment of various skills. The activity was intended to be delivered in-person for its second iteration in the spring of 2020; however, it transitioned to an asynchronous virtual activity with the transition to remote learning due to the COVID-19 pandemic.

The purpose of this study is to determine students’ perceptions of a PGx lab activity and its delivery through a virtual format.

## 2. Materials and Methods

The principles of PGx and management of drug therapy using PGx data are taught in the didactic curriculum during the fall semester of the second year. A clinical skills lab activity was developed for the following semester in the spring of the second year to reinforce these principles and allow students to navigate PGx information resources to develop and communicate recommendations for drug therapy. Upon completion of the PGx virtual learning activity, each student was expected to (1) learn to navigate pharmacogenomics-related databases; (2) demonstrate an awareness of the use and impact of pharmacogenomics within pharmacy and the health care system; and (3) effectively communicate pharmacogenomics-related pharmacotherapy and drug information recommendations using relevant pharmacogenomics-related databases.

**Figure 1:**
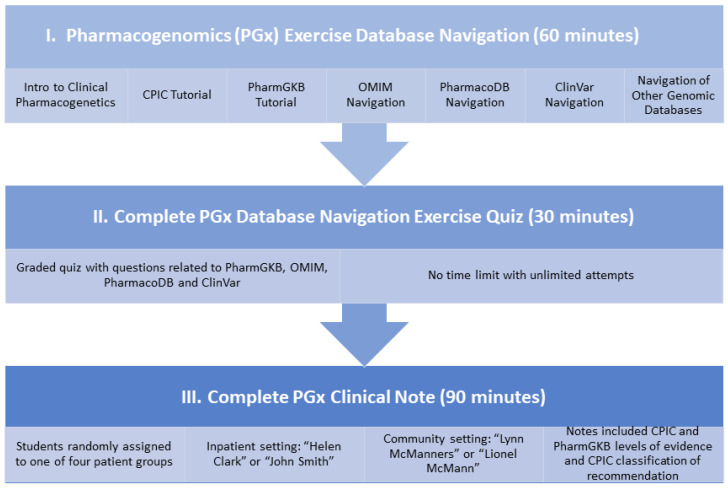
Flowchart and timing of virtual PGx learning activity.

The introduction to the PGx lab activity was conceptualized as a three-part activity (visualized in Figure 1). Part I was designed to give the students a 60 min, self-guided introduction to navigate through the most widely used databases for PGx information and guidelines, specifically, CPIC (https://cpicpgx.org/; accessed on 31 January 2022) and PharmGKB (https://www.pharmgkb.org/; accessed on 31 January 2022). Students were also exposed to several other PGx databases, specifically ClinVar (https://www.ncbi.nlm.nih.gov/clinvar/; accessed on 31 January 2022), Online Mendelian Inheritance in Man (OMIM; https://www.omim.org/; accessed 31 January 2022, PharmacoDB (https://pharmacodb.pmgenomics.ca/; accessed on 31 January 2022), and other genomic and precision medicine websites, including “All of Us” (https://allofus.nih.gov; accessed on 31 January 2022) and the “Alabama Genomic Health Initiative” (https://hudsonalpha.org/the-aghi/; accessed on 31 January 2022), through a short series of practice exercises. Students gained experience in navigation and search functions unique to each database by completing exercises that required them to search for a specific gene and/or other pre-determined phenotype and report their findings.

For Part II of the assignment, students took a graded quiz (constructed to take 30 min to complete) with an unlimited time and number of attempts to assess their familiarity with the websites introduced in Part I. Part III of the virtual PGx learning activity consisted of patient cases that challenged the students to utilize the PGx databases. The students evaluated a patient scenario identifying potential gene–drug interactions based on the patient’s genomic profile and evidence-based recommendations. Patient scenarios included two potentially actionable gene-drug interactions, a primary and a secondary, and several other non-genetic medication therapy errors commensurate with their level of didactic training. Part III patient cases were divided into inpatient and community settings to allow for communication adaptability to the target audience. The inpatient scenario allowed for pharmacist-to-physician exchange, while the outpatient scenario included a pharmacist-to-patient appropriate conversation. Four patient cases were constructed for each setting and given different patient names (inpatient: “Helen Clark” or “John Smith”; community: “Lynn McManners” or “Lionel McMann”). Each case included a primary drug with a potential actionable gene–drug paring and a secondary gene–drug interaction. The primary drug was defined as the drug that the predominance of the scenario was built around. The secondary drug was uniform across all four patient cases, but the genomic profile (i.e., patient genotypes) relative to that drug differed within each case. The students were given a history of present illness, past medical history, medication summary, and follow-up for each case. The patient’s PGx “genotype profile” and medication-related questions were included in the follow-up section. Specifically, students were tasked to provide written recommendations that included the patient’s PGx background information, potential gene–drug interactions and recommendations for their resolution, and any other recommendations for drug-related problems. The students had to provide support for their recommendations and to include the CPIC and level, PharmGKB levels of evidence, and the CPIC classification of recommendation. An anonymous, voluntary survey was sent to all students to capture their perceptions soon after completing the PGx virtual learning activity. This survey was sent through Samford University’s course management system, Canvas, and was available for ten days. The students were not provided with an incentive to participate in the survey. The survey asked for free-text responses to the following questions:

1. What did you learn from this pharmacogenomics (PGx) assignment?
2. What were your strengths during this learning activity?
3. What were your areas for improvement during this learning activity?
4. What did you like best about this PGx assignment?
5. What did you like least about this PGx assignment?
6. What recommendation(s) do you have for changing this PGx assignment?
7. What did you learn about the clinical application of pharmacogenomics from this learning activity?
8. If this learning activity is taught in the future, do you think it should be taught live (in person), synchronously (online instruction in real time), asynchronously (online instruction not in real time) or hybrid (blend of live and asynchronous)?
9. Please provide any additional comments about this assignment and/or suggestions for improving pharmacogenomics instruction at the McWhorter School of Pharmacy.

Survey responses were collected, and themes were identified among the responses.

## 3. Results

### 3.1. Survey Response

A total of 31 out of 113 students participated in the survey, giving a response rate of 27%. The study was conducted in accordance with the Declaration of Helsinki. The University’s Institutional Review Board approved this study as exempt since student responses were collected anonymously with no identifying information. The investigators gathered the student survey results and identified themes among the responses using content analysis for the purposes of improving teaching and learning in the virtual environment and as a guidance for other schools of pharmacy in the delivery of PGx lab activities using nontraditional teaching methodologies.

### 3.2. Themes and Supporting Quotes

Table 1 presents the major themes identified from each survey question, along with student comments that support these themes.

### 3.3. Student Preference for Delivery Format

**Figure 2:**
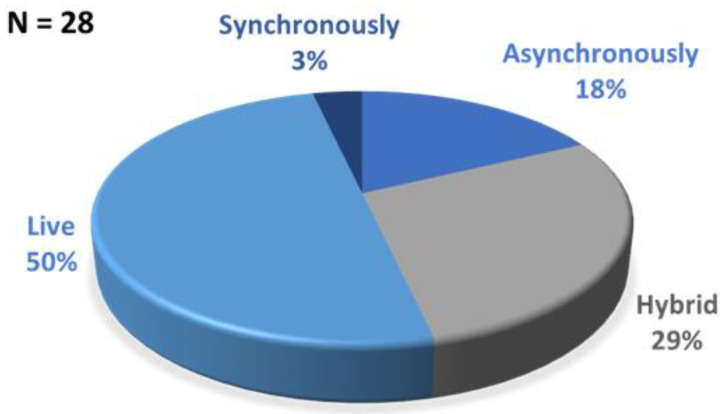
Responses to how should this learning activity be taught in the future (Question #8).

Figure 2 presents student responses to survey Question #8, which asked “If this learning activity is taught in the future, do you think it should be taught live (in person), synchronously (online instruction in real time), asynchronously (online instruction not in real time) or hybrid (blend of live and asynchronous)?”.

## 4. Discussion

Student responses revealed that there were things learned from this PGx activity, and suggested areas for improvement related to logistics. In general, students responded that they learned about the PGx databases and guidelines related to drug–gene interactions, and how PGx can be used in practice. Students also mentioned logistical challenges related to the time it took to complete the learning activity and a desire for clearer instructions and/or examples.

This virtual PGx learning activity took place in the spring of 2020, four weeks after the students began virtual learning during the COVID-19 pandemic. During this time, communication was erratic, and testing procedures were in flux. It is possible that the students would have experienced a smoother experience if, at the time, the faculty were more familiar with virtual learning and had developed communication techniques that translate well for virtual learners. Overall, this PGx learning activity represents a novel example of how to create an asynchronous, simulated PGx activity in a virtual learning environment.

There have been other studies that gauged student perceptions of a PGx activity, and of a PGx course. Patel and colleagues investigated students’ knowledge and perceptions of applying pharmacogenetics in a patient encounter using simulation [14]. Perception questions included confidence in their own as well as their team’s abilities to perform clinical activities using pharmacogenetic results. The results of the perception question related to their individual confidence improved in the post-simulation survey. Powers and colleagues investigated changes in knowledge, confidence, and skills of third-year pharmacy students in clinical pharmacogenetics following a laboratory session [15]. A confidence survey was administered to the students prior to and after the lecture upon which the session was based, and then again at the end of the semester. The post-lecture and post-lab results demonstrated statistically significant increases in confidence, and there were also significant increases in the post-lecture to post-lab results. Assem et al. surveyed pharmacy students before and after an intervention, whereby the students were given the opportunity to receive their PGx test results [16]. They reported increased confidence on each of the items related to conducting PGx counselling, and increased usefulness on each of the items relate to PGx testing.

Remsberg and colleagues investigated student perceptions of a pharmacogenomics course [17]. The pre-/post-course surveys asked students to rate confidence in their abilities to educate and manage patients using pharmacogenomics. The results of the post-course survey suggested that the course improved their confidence in their ability to educate and manage patients using pharmacogenomics. Marcinak and colleagues investigated the effectiveness of a required pharmacogenomics course, including perceived comfort and ability to apply the content in a clinical setting [18]. There were statistically significant increases in the items gauging perceived comfort and ability from the pre- to post-course surveys.

Coriolan and colleagues investigated perceptions and attitudes toward pharmacogenomics in pharmacy students from eight schools who were nearing graduation [19]. In contrast to other studies presented, this one did not investigate a specific learning experience, but rather perceptions from their overall training in pharmacogenomics. Given that there were multiple schools involved, the amount of pharmacogenomic content in their curricula varied from none to a required course. Responses related to clinical relevance were generally in agreement that pharmacogenomics is integral to the profession of pharmacy as well as to the practice of pharmacists.

These studies primarily investigated the students’ confidence in their abilities in pharmacogenomics. Comparing these to the current study, we did not specifically address confidence, and this was not one of the themes identified in student responses. Students did report, however, learning how to use resources needed to evaluate pharmacogenomic information and manage interactions. The students also reported learning the importance of pharmacogenomics in patient care.

Along with determining students’ perceptions of the lab activity, this current study also sought to determine students’ perceptions of the virtual format specifically. As schools move into a time where a variety of delivery methods are an option, it was important to gage the students’ preferences for delivery formats as there are times when each option is feasible. The majority of students in this study chose live delivery as opposed to hybrid, asynchronous, or synchronous. Themes emerged from the question of what students liked least about the assignment that indicated frustration with instructions and the time it took to complete the lab. This information can be useful for determining which types of content or processes are more conducive to certain delivery formats, as well as ways to improve an activity that would be delivered virtually.

The strengths of the current study are that qualitative methods allow respondents to give context to their responses as compared to quantitative results. Additionally, questions gathered various aspects of students’ preferences as well as what they learned. Lastly, information regarding format for teaching the activity in the future can be useful. The limitations include a lower response rate, and the overall timing of the activity. In the spring of 2020, the investigators were novices at developing and implementing virtual activities so there are elements of the frustrations expressed by respondents that may no longer be applicable as we have gained experience in this.

Future iterations of this activity could include modifications to instructions and timing of the activity to allow it to be more conducive to a virtual format. This would help to determine whether the virtual format was based on logistics or whether the activity is truly best delivered in person.

## Figures and Tables

**Table 1:** Survey question themes and supporting quotes.

Survey Question	Theme(s)	Student Comments
1. What did you learn from this PGx assignment?	Databases and information	“I learned how to use databases to access pharmacogenomic drug interactions.” “I learned what pharmacogenomics databases were available and how to use them.” “I learned to be able to proficiently navigate the PGx databases, and how to read and interpret the CPIC guidelines.” “How to use various PGx resources and how to access information on various drug–gene interactions.”
2. What were your strengths during this learning activity?	Navigate websites	“My strength was conducting the search for the genes and drug interactions. It was easy for me to navigate the websites needed to complete the assignment.” “I feel that my strengths in this activity were being able to navigate and find the other guidelines that were needed to make recommendations and be able to critically think about what drug(s) could be optimized for the patient.” “Finding research on the CPIC website to figure out if patient needs to take different medication based on their genotyping.”
3. What were your areas for improvement during this learning activity?	Long, time, note, video, websites	“The note was a little confusing and I was unsure of exactly what to do.” “I could improve upon my knowledge of PGx. Most of the information was unfamiliar to me.” “Need to familiarize with websites more.” “I feel as though the lab could have been explained more. It was also really long considering this time of online learning.” “It took me twice as long as lab normally lasts to complete this activity.”
4. What did you like best about this PGx assignment?	Patient, guidelines, recommendations, databases	“Learning about CYP metabolism and applying new information to a patient case.” “Learning that there is evidence behind why some drugs work for some people but not all even though the disease state may be the same.” “I enjoyed learning about all the databases I can utilize when treating a patient.” “I liked the case scenario. It is definitely a situation that we would encounter as practicing pharmacists and this practice would help develop the skills to properly respond when it does occur.” “I enjoyed the puzzle aspect of the assignment. I liked following the clues of the genetic testing results to guidelines to making recommendations that could benefit the patient in multiple ways.”
5. What did you like least about this PGx assignment?	Quiz, time, answers, instructions	“It was extremely long and I was confused by the directions.” “Having minimal guidance throughout the lab and having to figure out/troubleshoot problems on my own. This was very discouraging because the lab took me twice as long due to this.” “I felt very unprepared and confused about the instructions, it was very lengthy.” “I thought the length due to the number of medications he/she was taking and genes that were looked at.” “This took an extended amount of time. I would suggest that next year this be given in January or February when there is a lull in lab activities. We have so much going on right now, and even if we were not living and learning under quarantine, we would still be stressed with the last exams of our two major classes around this time and finals looming. It’s great learning experience. I just wish it had been when I was not so busy and stressed. Additionally, the Canvas quiz was not really necessary in my opinion. We have enough background knowledge on CYP enzymes and polymorphisms by spring of P2 year to just do the assignment without it.”
6. What recommendation(s) do you have for changing this PGx assignment?	Instructions, time, note, lab	“Instructions on navigating the website should be clearer to cut back on time performing web searches.” “A review of terminology before the lab. In class assignment and in groups.” “If there is a way to incorporate this assignment with EHR Go I think it would improve the delivery of this assignment.” “It was difficult as an online module. I believe many issues would be resolved by in person instruction like the lab was initially planned.” “I would try to make the assignment just a bit shorter. And perhaps make the instructions a little more clear like if we needed to include recommendations on therapy that was not one of the results of the genome analysis.”
7. What did you learn about the clinical application of PGx from this learning activity?	Important, certain, medications, different genes, patients	“It helped me integrate pharmacogenomics into an MTM like scenario.” “I learned that it is important for some medications to do genetic testing before prescribing a medication because it may not work at all in the patient, or it may need a dose adjustment due to certain mutations in genes.” “I learned that individualized medicine is a necessary development and understanding that patients may vary in their metabolizing capability is important in tailoring their pharmacotherapy.” “I learned about different resources that can be used to help modify treatment for patients based on their specific genes.”
8. Please provide any additional comments about this assignment and/or suggestions for improving PGx instruction at the McWhorter School of Pharmacy.	Time, example, semester	“I would have enjoyed an example note.” “If you could have the guest speaker there during lab that is a clinical pharmacist working in pharmacogenomics to help navigate the different websites, that would be a great experience.” “I would like to see more pharmacogenomics explicitly included in the curriculum.”
